# Characterization of serous retinal detachments in uveitis patients with optical coherence tomography

**DOI:** 10.1007/s12348-012-0084-8

**Published:** 2012-06-02

**Authors:** Annamieka Simmons-Rear, Steven Yeh, Brian T. Chan-Kai, Andreas K. Lauer, Christina J. Flaxel, Justine R. Smith, James T. Rosenbaum, Eric B. Suhler

**Affiliations:** 1 Casey Eye Institute, Oregon Health and Science University, Portland, OR USA; 2 Emory Eye Center, Emory University, Atlanta, GA USA; 3 Cullen Eye Institute, Baylor College of Medicine, Houston, TX USA; 4 Veterans Affairs Medical Center, Portland, OR USA

**Keywords:** Optical coherence tomography, OCT, Uveitis, Serous retinal detachment, Macular edema, Vogt-Koyanagi-Harada syndrome, Sarcoidosis, Acute posterior multifocal placoid pigment epitheliopathy, Pars planitis, Hypotony maculopathy, Choroidal neovascularization

## Abstract

**Objectives:**

To determine the prevalence of serous retinal detachments (SRD) using optical coherence tomography (OCT) in a large database of patients with uveitis from a tertiary referral setting, to describe clinical features of patients with SRD, and to ascertain retinal architectural features found in association with SRD.

**Main outcome measures:**

Prevalence of SRD in uveitis patients imaged with OCT, correlation of visual acuity with SRD, anatomic subtypes of uveitis identified, and association of SRD with various subtypes of macular edema (focal and diffuse) and retinal architectural abnormalities.

**Design:**

Retrospective, single-setting cross-sectional study of all OCTs in a digital imaging base ordered on patients from a tertiary referral uveitis clinic between July 2006 and March 2008.

**Results:**

SRD were identified in 17 of 111 uveitis patients (15 %) reviewed; bilateral SRD were found in 5 of 17 patients (29 %). Intermediate uveitis was the most common disease association (47 %), but other conditions identified included Vogt-Koyanagi-Harada syndrome, multifocal choroiditis/panuveitis, and sarcoidosis. Retinal architectural features identified in association with SRD included focal macular edema (59 %), diffuse macular edema (50 %), any intraretinal edema (77 %), both diffuse and focal macular edema (32 %), and retinal pigment epithelial alteration (27 %). Moderate or severe visual impairment, defined as visual acuity 20/50 or poorer was seen in 71 % of patients with SRD. Poorer visual acuity was correlated with increased central subfield thickness in patients with SRD (*r* ^2^ = 0.41, *p* < 0.001).

**Conclusion:**

SRD were present in 15 % of the uveitis patients reviewed. Moderate to severe vision impairment was present in the majority of eyes (71 %) with SRD. Diffuse macular edema and focal cystoid macular edema were the OCT features most commonly associated with SRD. Intermediate and panuveitis were the most common anatomic sites of inflammation. A variety of pathogenic mechanisms, both inflammatory and non-inflammatory, may be involved in SRD in uveitis patients; identification of the precise mechanism is important for appropriate therapy.

## Introduction

The term uveitis is used to describe a heterogenous group of inflammatory diseases having in common intraocular inflammation. Uveitis is recognized as a leading cause of blindness in the Western world that differentially affects younger, working-age adults and children and causes vision loss both due to direct effects and indirectly via secondary anatomic complications such as cystoid macular edema. Macular edema is present in up to 33 % of uveitis patients and is responsible for visual impairment or blindness in 59 % of patients with panuveitis and 85 % of patients with intermediate uveitis [1].

Our ability to resolve retinal architectural features has been revolutionized by the advent of optical coherence tomography (OCT), a highly sensitive method of obtaining cross-sectional imaging of the retina to diagnose, follow, and characterize macular edema in uveitis and other disease states. OCT is now the most widely used modality for detecting macular disease [2–5]. With this improved ability to characterize macular ultrastructure, three subtypes of macular edema have been identified [3, 5]. The first two, diffuse and cystoid macular edema, are caused by intraretinal fluid accumulation in the neurosensory retina. The third, serous retinal detachment (SRD), is characterized by fluid accumulation in the potential space between the macula and underlying retinal pigment epithelium. Besides its presence in patients with macular edema, neurosensory retinal detachment may also result from other mechanisms independent of inflammation-associated macular edema (e.g., choroidal neovascularization).

Prior studies have shown that SRDs are found in 15–20 % of uveitis patients with macular edema [3, 5], but there is conflicting literature as to the correlation between the presence of SRD and decreased visual acuity. The prognostic significance of SRD has not been established in the literature and is difficult to assess given the heterogeneous nature of secondary causes, including Vogt-Koyanagi-Harada syndrome (VKH) [6, 7], toxoplasmosis [8], Behcet’s disease [9, 10], and juvenile idiopathic arthritis.

Syndromes associated with uveitis, such as VKH, often demonstrate retinal pigment epithelial (RPE) damage in addition to serous detachments. The destruction of the RPE layer in association with serous detachments has been postulated to be a result of inflammation and ischemic damage [2]. Our study aimed to investigate the degree of visual impairment present in eyes in our series with SRD and to examine the spectrum of retinal morphological changes present with SRD in association with a variety of disease processes.

## Methods

We surveyed all OCTs and associated patient records from patients from the Uveitis and Immunology service of the Casey Eye Institute performed for diagnostic and management purposes between July 2006 and March 2008. Two separate investigators (A.S-R and S.Y.) evaluated the OCTs for the presence and type of macular edema, including diffuse and cystoid macular edema and SRDs, and other OCT features. A third investigator (B.C.K.) adjudicated any differences in the presence of SRD and other abnormalities identified by OCT. SRD were defined as an elevation of the neurosensory retina with an optically clear space between the retina and the RPE layer. Diffuse macular edema (DME) and cystoid macular edema (CME) were defined according to previously described patterns of macular edema in uveitis (i.e., DME, CME, SRD) [3–5]. Fluorescein angiograms and fundus photography were also reviewed in cases where SRDs were identified. Because our study included patients with suspected macular disease, some individuals with SRD in the absence of macular edema were also captured in our imaging cohort. Of note, one patient with central serous retinopathy was identified in our review. Because this finding was considered unrelated to underlying ocular inflammation, the patient was excluded from the statistical analysis.

Our study protocol was Institutional Review Board-approved, and all research conformed to the Association for Research in Vision and Ophthalmology statement on human research. Specific medical record data gathered for each patient included age, gender, uveitis anatomic classification based as defined by Standardization of Uveitis Nomenclature criteria [11], laterality, Snellen visual acuity, associated systemic diagnoses, and the presence of other local disease processes potentially contributing to the SRD (e.g., concomitant choroidal neovascular membrane).

### Optical coherence tomography protocol

OCT measurements were obtained using the Stratus OCT3 (software ver. 5.0), a time-domain OCT instrument (Carl Zeiss, Meditec, Inc., Dublin, CA). Scanning was performed using the fast macular thickness map protocol, consisting of six evenly spaced 6-mm radial lines for quantitative measurements; each line consisted of 128 A-scans, intersecting at the fovea, for a total of 768 sampled points with a scanning time of 1.9 s. Central 1-mm subfield thickness (Area A1) was recorded using the manufacturer’s imaging software. In instances when automated central macular thickness could not be computed due to marked thickening, the caliper mode on the Stratus OCT was used for a manual center point thickness measurement. High-resolution vertical and horizontal cross-hair patterns (9–3 o’clock and 6–12 o’clock) were also obtained for assessment of evidence of overlying macular edema (cystoid versus diffuse), retinal pigment epithelium scar, and foveal atrophy.

### Statistical analysis

Descriptive statistics were performed using Microsoft Excel (Microsoft, Redmond, WA). Inferential statistics were performed using GraphPad Prism (La Jolla, CA). Patients were classified according to the level of visual impairment for descriptive purposes. Mild visual impairment was defined as Snellen visual acuity of 20/25 to 20/40. Moderate visual impairment was defined as visual acuity poorer than or equal to 20/50 and better than 20/200. Severe visual impairment was defined as visual acuity of 20/200 or poorer. Snellen visual acuity was converted to the log of the minimal angle of resolution (logMAR) visual acuity using the formula logMAR VA = −log (decimal equivalent of Snellen visual acuity). Linear regression and two-tailed Pearson correlation analysis were performed to determine whether a relationship existed between logMAR visual acuity and central subfield thickness. An alpha of 0.05 was considered statistically significant.

## Results

OCTs from a total of 111 patients were reviewed, and of these patients, 17 (15 %) patients demonstrated SRD by OCT; five patients were bilaterally affected, resulting in a total of 22 affected eyes. Twelve patients (71 %) were female, and the mean age of patients was 39.8 years (range, 9–64 years). The mean visual logMAR visual acuity ± standard deviation was 0.718 ± 0.779 (Snellen visual acuity equivalent 20/104). Eleven of 22 eyes (50 %) showed moderate visual impairment, defined as VA ≤ 20/50 and > 20/200. Five eyes (23 %) showed severe visual impairment, VA ≤ 20/200. The demographic characteristics and visual acuity of patients are summarized in Table 1.

**Table 1:** Characteristics of uveitis patients with SRD

Total no. of patients	17
Male (%)	5 (29)
Female	12 (71)
Laterality	
Unilateral (%)	12 (71)
Bilateral	5 (29)
Mean age, years (range)	39.8 (9–64)
Visual acuity, logMAR ± SD (Snellen)	0.718 ± 0.779 (20/104)
No. of eyes at degree of visual impairment (%)	22 (100)
Mild 20/25-20/40	6 (27)
Moderate 20/50–20/160	11 (50)
Severe 20/200 or worse	5 (23)
Anatomic location of uveitis	
Anterior (%)	0 (0)
Intermediate	8 (47)
Posterior	1 (6)
Panuveitis	8 (47)
Associated syndrome	No. of patients (%)
Idiopathic	9 (41)
Vogt-Koyanagi-Harada (VKH)	2 (12)
Postoperative	2 (12)
Sarcoidosis	1 (6)
Acute posterior multifocal placoid pigment epitheliopathy (APMPPE)	1 (6)
Hypotony maculopathy	1 (6)
Multifocal choroiditis/panuveitis-associated choroidal neovascularization	1 (6)

*logMAR* log minimal angle of resolution, *VA* visual acuity, *SD* standard deviation

Identified secondary diagnoses are summarized in Table 1, including VKH (two), sarcoidosis (one), acute posterior multifocal placoid pigment epitheliopathy (one), hypotony maculopathy (one), panuveitis-associated choroidal neovascular membrane (one), and post-operative inflammation following cataract removal (two).

Anatomic diagnoses included intermediate uveitis (eight patients, 47 %), posterior uveitis (one patient, 6 %), and panuveitis (eight patients, 47 %). Patients with anterior and intermediate uveitis were classified as intermediate uveitis; no patients with SRD with isolated anterior uveitis were identified (Table 2).

**Table 2:** Systemic and ophthalmic diagnoses and OCT features of uveitis patients with SRD

Patient no.	Age/gender	Eye	Anatomic diagnosis	Diagnosis	Concomitant ophthalmic diagnosis	Visual acuity	DME	CME	RPE change	CST
1	45/F	OS	Anterior and intermediate	Idiopathic		20/30	+	+	+	546
2	49/F	OD	Anterior and intermediate	Idiopathic		20/50		+		554
		OS				20/50		+		455
3	37/M	OD	Anterior and intermediate	Idiopathic		20/60	+	+		372
4	46/F	OD	Anterior and intermediate	Idiopathic		20/25				194
5	59/F	OS	Anterior and intermediate	Chronic, postoperative uveitis		20/200	+			715
6	64/F	OD	Intermediate	Idiopathic		20/70	+	+		645
		OS				20/70	+	+		463
7	11/M	OS	Intermediate	Pars planitis		20/50		+		492
8	29/M	OS	Intermediate	Chronic, postoperative uveitis		20/400	+		+	279
9	12/F	OD	Panuveitis	Idiopathic		20/50	+	+		533
10	42/F	OS	Panuveitis	Idiopathic		20/30			+	210
11	9/F	OD	Panuveitis	Idiopathic		20/40		+		459
		OS				20/40		+		532
12	53/M	OS	Panuveitis	Idiopathic	CNVM	20/50	+		+	322
13	45/F	OS	Panuveitis	MFC/PU	Hypotony maculopathy	CF	+	+		1718
14	57/F	OD	Panuveitis	Sarcoidosis		20/50		+	+	311
		OS				20/100			+	235
15	30/M	OD	Panuveitis	VKH		20/30				285
16	39/F	OD	Panuveitis	VKH		CF	+	+		1168
		OS				CF	+			443
17	49/F	OD	Posterior	APMPPE		20/50				307

*VKH* Vogt-Koyanagi Harada disease, *CF* counting fingers, *CSR* central serous retinopathy, *CNVM* choroidal neovascular membrane, *MFC*/*PU* Multifocal choroiditis/panuveitis syndrome, *DME* diffuse macular edema, *CME* cystoid macular edema, *RPE* retinal pigment epithelium, *PED* pigment epithelial detachment

**Fig. 1:**
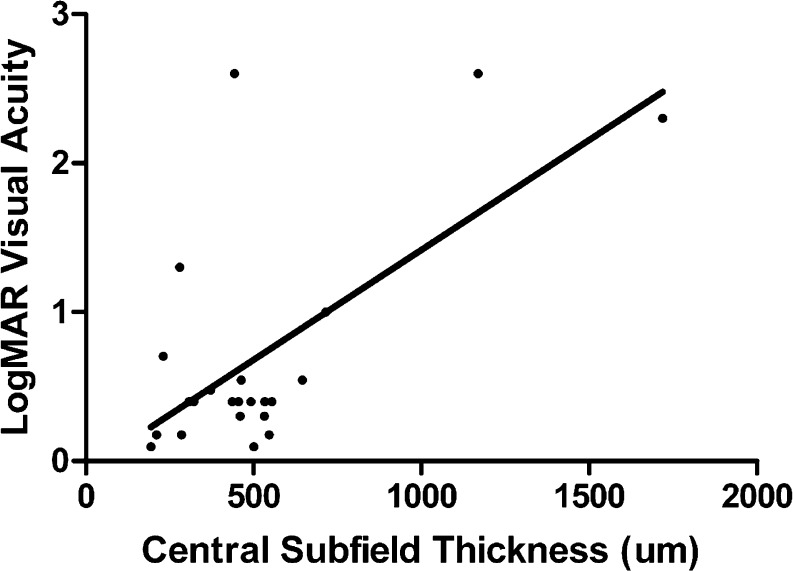
LogMAR visual acuity versus central subfield thickness in patients with SRD. Linear regression curve shows a positive slope and two-tailed Pearson correlation analysis shows *r* ^2^ = 0.41 (*p* = 0.0009)

The mean central subfield thickness (CST) ± standard deviation was 519 μm ± 341 (range, 210–1718 μm). Linear regression and two-tailed Pearson correlation analysis evaluating the relationship of logMAR visual acuity and CST showed a statistically significant positive correlation between poorer logMAR visual acuity and increased CST (Fig. 1, *r* ^2^ = 0.41, *p* = 0.0009).

Retinal architectural abnormalities identified on OCT, which were associated with SRD, are summarized in Table 3. These architectural abnormalities included diffuse macular edema in 11 eyes (50 %), focal cystoid macular edema in 13 eyes (59 %), any intraretinal edema (diffuse or focal) in 17 of 22 eyes (77 %), both focal and diffuse macular edema in seven eyes (32 %), and retinal pigment epithelium abnormalities in six eyes (27 %).

**Table 3:** Retinal architectural findings associated with SRD

Retinal architectural finding	No. of eyes (%)
Total	22 (100)
Any intraretinal edema (diffuse or cystoid)	17 (77)
Diffuse macular edema	11 (50)
Focal cystoid macular edema	13 (59)
Both diffuse and focal macular edema	7 (32)
Retinal pigment epithelium change	6 (27)

To illustrate the spectrum of disease processes identified in this uveitis patient population, we have summarized herein four illustrative cases of patients identified in this series with SRDs of varying etiologies, management strategies, and prognostic implications.

### Illustrative cases

**Fig. 2:**
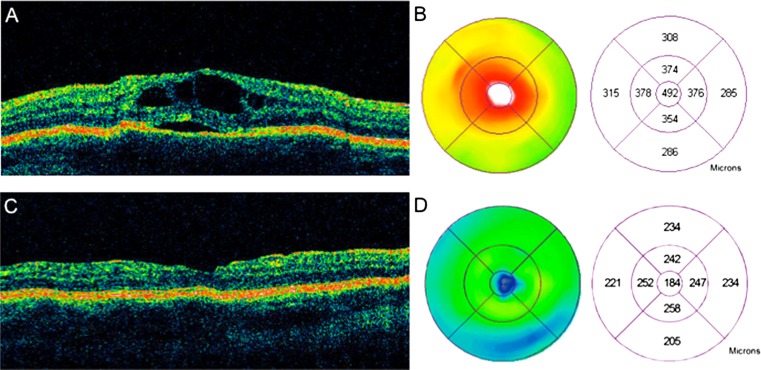
OCT and macular thickness map of a SRD in an 11-year-old patient with pars planitis. Focal cysts overlying a SRD are seen **a** with central macular thickening and a central subfield thickness of 492 um (**b**). Following an oral prednisone taper and methotrexate immunosuppression, the SRD and focal cystoid macular edema have resolved (**c**) with an improvement in visual acuity, decrease in visual symptoms, and restoration of normal macular thickness on the macular thickness with a central subfield thickness of 184 um (**d**)

Patient 7 is an 11-year-old male with pars planitis oculus uterque (OU; both eyes), status post-pars plana vitrectomy/cryotherapy OU and history of disease remission on methotrexate who presented with increasing floaters and blurred vision OU. His visual acuities were 20/40 oculus dexter (OD; right eye) and 20/50 oculus sinister (OS; left eye). Slit lamp examination was quiet OD and revealed 1+ anterior chamber inflammation OS and 1+ vitreous cell OS. Dilated funduscopic examination showed peripheral cryotherapy scars OU and cystoid macular edema OS. OCT demonstrated a foveal SRD with overlying cystoid macular edema with overlying focal cystoid macular edema (Fig. 2).

Methotrexate 15 mg/ week and slow prednisone taper were prescribed. Four weeks thereafter, the visual acuity improved to 20/40 OS with an improvement in inflammation to trace anterior chamber cell and trace vitreous cell OS. The focal cystoid macular edema and SRD had completely resolved (Fig. 2). At 21 months follow-up, the visual acuity was stable at 20/30 OS on methotrexate therapy, and no evidence of recurrent inflammation was seen.

**Fig. 3:**
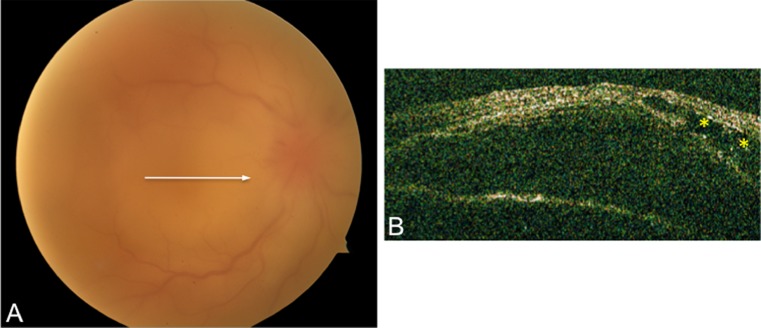
Fundus photograph and OCT of a patient with acute VKH disease. Fundus photograph show vitreous haze, optic disc edema, and multiple exudative detachment involving the posterior pole (**a**). OCT reveals dome-shaped SRD with intraretinal, focal cystoid edema OD (**b**)

Patient 17 is a 39-year-old Latin American woman who presented with blurred vision, headaches, tinnitus, and panuveitis with multiple exudative retinal detachments OU of 3 weeks duration. She had been treated unsuccessfully with topical prednisolone acetate 1 % and atropine 1 % BID. Visual acuity on presentation was counting fingers OU. Slit lamp examination showed 3+ anterior chamber cell OU and 2+ vitreous cells OU. Detailed funduscopic features were difficult to appreciate secondary to 2+ vitreous haze OU. There was severe optic disc edema OU and multiple neurosensory retinal detachments OU, which were demonstrated by OCT (Fig. 3). Acute Vogt-Koyanagi-Harada’s disease was diagnosed, and methylprednisolone 1 mg/ kg × three doses was administered intravenously followed by prednisone 1 mg/ kg/ day orally. Prednisone was slowly tapered off, and mycophenolate mofetil 1 g twice daily was started with maintenance of clinical disease remission. At 20 months follow-up, the patient’s visual acuity was 20/30 OD and 20/40 OS. Funduscopic examination showed a typical “sunset-glow” fundus of VKH from choroidal depigmentation and resolution of the SRDs.

## Discussion

In this study, we identified SRDs in 15 % of all patients with uveitis who underwent time-domain OCT imaging due to suspected macular pathology. Intermediate and panuveitis were the most common anatomic sites of inflammation, which is consistent with prior research evaluating uveitic macular edema [1]. Moderate or severe visual impairment was identified in 71 % of patients in this series. We also found that increased CST was correlated with poorer logMAR visual acuity in patients with SRD. Whether decreased vision was a direct result of the SRD is difficult to ascertain due to high prevalence of concurrent intraretinal macular edema, which may also cause reduced visual acuity. Other factors including photoreceptor integrity, presence or absence of foveal atrophy, and chronicity of macular edema likely contributed to visual impairment, but these factors were not specifically addressed in the context of this study.

Diffuse macular edema and focal cystoid macular edema were the OCT features most commonly associated with SRD. The focal subtype of macular edema was most commonly identified while the diffuse subtype was also seen in a large proportion of patients. Patients with intermediate uveitis and cystoid macular edema comprised the majority of patients with OCT evidence of SRD and is consistent with prior series of patients with macular edema in tertiary referral settings [3, 12].

A variety of pathogenic mechanisms appear to be involved in the development of SRD in the uveitis population. Macular edema, the retinal architectural feature and clinically identifiable feature typically prompting OCT evaluation, is caused by inflammatory disruption of the normal permeability of the blood–retinal barrier.

Uveitis therapy with corticosteroids or other immunosuppressive medications leads to reduction in inflammation, restoration of vascular permeability, and subsequent resolution of the SRD and macular edema. However, because of the variety and diversity of etiologies associated with uveitis, other mechanisms are likely involved as well.

In VKH, breakdown of the blood–retinal barrier from iris and ciliary body inflammation is likely compounded by choroidal inflammation, overlying RPE injury and poor RPE pump function leading to the massive exudative detachments found in this disease process. In patients with multifocal choroiditis and punctate inner choroidopathy, subretinal fluid may accumulate following the formation of a choroidal neovascular complex, a consequence of both inflammatory and angiogenic pathways. Increased levels of vascular endothelial growth factor (VEGF) have been identified in the aqueous humor of uveitis patients [13], and recently, anti-VEGF therapy with and without concomitant immunosuppressive medication has been successfully used for the treatment of uveitis-associated choroidal neovascular membrane (CNVM) [14, 15].

Within this series of patients, the specific therapy required to treat the process and associated SRD varied according to the disease process. Specifically, corticosteroid treatment was indicated for treatment of SRD secondary to inflammation as in intermediate uveitis and VKH; however, in patients with SRD due to CNVM, anti-VEGF and anti-inflammatory medications were required. Central serous retinopathy, although not specifically addressed in the context of this study, is another important etiology to consider, as corticosteroid use is nearly ubiquitous in the uveitis patient population and requires corticosteroid withdrawal to expedite its resolution.

The limitations of this study include the retrospective, cross-sectional nature of the study. Any study from tertiary referrals centers is prone to ascertainment bias, potentially skewing the proportion of uveitis patients with SRD. Conversely, some patients with advanced uveitis and severe vitritis and retinitis were excluded by the nature of the severity of their disease, which may cause significant media opacity precluding OCT imaging. Only a subset of patients with uveitis was characterized by OCT, and this subset mostly included patients with suspected macular edema. Lastly, our study exclusively used time-domain OCT, which utilizes 400 axial A-scans per second to achieve an axial resolution of 10 μm, whereas newer spectral domain OCT technology utilizes 20,000 A-scans per second to achieve a resolution approaching 5 μm. Moreover, because the retinal segmentation algorithms of spectral domain (SD)-OCT may result in greater macular thicknesses when compared with the algorithms of the time domain-OCT protocol used in this investigation, the CST measured in this study may be underestimated in patients with SRD.

Despite these limitations, we were able to identify specific uveitis syndromes in which SRD was a key clinical feature and described OCT characteristics valuable for planning future longitudinal studies employing SD-OCT strategies in the measurement of structural outcomes. Future prospective studies are needed and could provide more objective data by comparing the presence of SRDs in uveitis patients without macular edema to those with macular edema and might better define the visual prognostic significance of different subtypes of uveitic macular edema and their response to therapy.
